# Enhanced Temporal Coupling between Thalamus and Dorsolateral Prefrontal Cortex Mediates Chronic Low Back Pain and Depression

**DOI:** 10.1155/2021/7498714

**Published:** 2021-10-08

**Authors:** Hong Li, Qiaoyan Song, Ruya Zhang, Youlong Zhou, Yazhuo Kong

**Affiliations:** ^1^CAS Key Laboratory of Behavioral Science, Institute of Psychology, Beijing 100101, China; ^2^Department of Psychology, University of Chinese Academy of Sciences, Beijing 100049, China; ^3^Shanxi Key Laboratory of Artificial Intelligence Assisted Diagnosis and Treatment for Mental Disorder, First Hospital of Shanxi Medical University, Taiyuan 030000, China; ^4^Department of Psychiatry, First Hospital/First Clinical Medical College of Shanxi Medical University, Taiyuan 030000, China; ^5^Department of Pain, The Third Affiliated Hospital of Henan University of Traditional Chinese Medicine, Zhengzhou 450008, China

## Abstract

Numerous neuroimaging studies have demonstrated that the brain plasticity is associated with chronic low back pain (cLBP). However, there is a lack of knowledge regarding the underlying mechanisms of thalamic pathways for chronic pain and psychological effects in cLBP caused by lumbar disc herniation (LDH). Combining psychophysics and magnetic resonance imaging (MRI), we investigated the structural and functional brain plasticity in 36 patients with LDH compared with 38 age- and gender-matched healthy controls. We found that (1) LDH patients had increased psychophysical disturbs (i.e., depression and anxiety), and depression (Beck-Depression Inventory, BDI) was found to be an outstanding significant factor to predict chronic pain (short form of the McGill Pain Questionnaire, SF-MPQ); (2) the LDH group showed significantly smaller fractional anisotropy values in the region of posterior corona radiate while gray matter volumes were comparable in both groups; (3) resting state functional connectivity analysis revealed that LDH patients exhibited increased temporal coupling between the thalamus and dorsolateral prefrontal cortex (DLPFC), which further mediate the relationship from chronic pain to depression. Our results emphasized that thalamic pathways underlying prefrontal cortex might play a key role in regulating chronic pain and depression of the pathophysiology of LDH.

## 1. Introduction

Chronic low back pain (cLBP) is one of the most common reasons for adults to visit the clinic [1, 2]. Clinically, lumbar disc herniation (LDH), which is mainly caused by the degeneration of the lumbar disc annulus or the external pressure force burdened on the disc, is an important cause of cLBP [3]. LDH patients are clinically characterized by extreme pain and emotional comorbidities, such as anxiety and depression, which seriously diminishes the patient's quality of life [4, 5]. Unfortunately, current treatment effect for LDH is unsatisfactory [6]. This is at least partial due to the limited understanding of the underlying mechanisms of chronic pain and psychological effects of LDH.

Neuroimaging studies have concluded that central nervous system is engaged in the development of cLBP, and the brain of chronic pain patients is continuously processing spontaneous pain by integrating information between multiple brain regions, mainly including primary somatosensory cortex (S1), anterior cingulate cortex (ACC), thalamus, insula, and periaqueductal gray (PAG) [6–8]. In particular, numerous studies suggest a critical role of the thalamus in chronic pain processing [9–11]. Structurally, Apkarian et al. found that gray matter density was reduced in the right thalamus and was closely correlated with the different patterns of pain characteristics for neuropathic and nonneuropathic cLBP, suggesting that the pathophysiology of chronic pain includes the thalamocortical processes [5]. Functionally, Llinás et al. identified that a common mechanism is operant, that is, the abnormal low-frequency oscillations of the thalamocortical network associated with dysregulations and symptoms attributed to various chronic pain conditions [12]. In line with this, studies using functional magnetic resonance imaging (fMRI) have indicated that low-frequency oscillations and abnormal connectivity of the thalamocortical networks are the basis for persistent pain [9, 13].

Notably, among the thalamocortical linkage, some researchers emphasized the functional connectivity of the thalamus with prefrontal cortex (PFC), possibly reflecting individual differences in pain modulation. It is well documented that the mediodorsal thalamus employs a role in the affective dimension of pain, and deficits of the thalamus and PFC coupling have been well documented in some psychiatric conditions, including depression disorder and schizophrenia [14]. Chronic pain and depression are highly intertwined clinically, which could result in longer duration of symptoms and poor prognosis [15]. Given their comorbidity, common mechanisms have been suggested in pain and depression [16]. Many brain regions, such as the thalamus, medial prefrontal cortex, and amygdala, are involved in the central modulation of chronic pain and depression, suggesting a common mechanism for dysregulation of emotional and reward processing [17, 18]. However, until now, the exact neurobiological mechanism of pain and depression remains unknown, which needs to be further clarified. Investigating how thalamic pathways modulate chronic pain and depression in LDH is essential for facilitating the targeted intervention options for patients.

In the current study, combining psychophysics with MRI techniques, we investigated the structural and functional brain alterations in patients with LDH compared with age- and gender-matched healthy controls (HCs). We related these brain alternations with pain intensity as well as pain-related emotional comorbidities in the patient group. We hypothesized that LDH patients (1) exhibited increased psychophysical problems and (2) had structural and functional thalamic abnormalities, which is significantly related to its pain intensity and pain-related emotional comorbidities.

## 2. Materials and Methods

### 2.1. Participants

A total of 36 right-handed LDH patients (25 males, mean age 45.11 ± 10.57 years) and 38 age- and gender-matched right-handed HCs (28 males, mean age 43.68 ± 11.86 years) participated in the study. All patients met the following inclusion criteria: (1) diagnostic and radiological evidence (CT or MRI) for LDH was confirmed by 3 experienced clinicians, (2) radiating pain > 3 score (assessed by visual analogue scale (VAS)); and (3) duration of pain > 3 months. All enrolled patients did not take any painkillers at least one week before the MRI scan. None of the participants had a past or current diagnosis of any psychiatric or major neurological illness. Participants signed the informed consent prior to the experiment on the premise of fully understanding the content of the experiment. The local ethics committee at the Third Affiliated Hospital of Henan University of Chinese Medicine approved the study. All participants were recruited from 2018 to 2019. For the demographic data, group differences in age and education level were evaluated using independent-sample *t*-tests, and differences in gender were assessed using chi-square test.

### 2.2. Pain Characteristics

Pain characteristics were assessed using the short form of the McGill Pain Questionnaire (SF-MPQ) [19] and pain sensitivity questionnaire (PSQ) [20], and all participants completed the assessments before the MRI scan. The SF-MPQ comprises (1) a pain rating index (PRI), which includes 15 descriptors, and the range is divided into 4 levels from 0 (none) to 3 (severe); (2) a present pain intensity (PPI) index ranging from 0 (no pain) to 5 (unbearable pain), which evaluates the present pain intensity; and (3) a visual analogue scale (VAS) to assess the average daily pain intensity in the past two weeks. The SF-MPQ total score is the sum of these three subscales. PSQ consists of 17 items. It has been widely used to evaluate the participants' pain sensitivity and has been proven to have good reliability and validity [20].

### 2.3. Psychophysical Characteristics

All participants completed psychological questionnaires to assess depression (Beck-Depression Inventory, BDI) as well as state and trait anxiety (State-Trait Anxiety Inventory, STAI) [21]. In addition, debriefing of participants includes questionnaires on sleep quality (Pittsburgh sleep quality index, PSQI) and pain vigilance (pain vigilance and awareness questionnaire, PVAQ) [22, 23]. The pain and psychophysical characteristics' differences between LDH patients and HCs were performed by using independent-sample *t*-tests. For LDH patients, Pearson's correlation analyses were adopted to assess the relationship between pain intensity, psychological variables, and thalamus-based functional connectivity.

In order to assess the joint influence psychophysical factors on chronic pain, a multiple linear regression analysis based on a forward stepwise selection procedure was performed. In this analysis, the severity of chronic pain (overall scores of SF-MPQ) was used as the dependent variable, and the sleep disturbances (PSQI) as well as psychological characteristics (SAI, TAI, BDI, and PVAQ) were identified as explanatory variables. First, the forward stepwise selection procedure includes the explanatory variable that could significantly explain the dependent variable (*p* < 0.05). Then, the procedure adjusts by repeatedly adding other explanatory variable (if any) that could significantly improve the fitting of the model (*p* < 0.05), until no one could improve the model. Before performing multiple linear regression analysis, all variables were examined on deviation from normality using Kolmogorov-Smirnov test (SPSS, IBM, Mac version 23.0.0), and no serious deviation from the normality was observed for any variable (*p* > 0.05).

### 2.4. MRI Acquisition

All MRI images were collected using a 3T Siemens Skyra scanner at the Third Affiliated Hospital of Henan University of Chinese Medicine. Structural MRI data were acquired using a 3D magnetization-prepared rapid gradient-echo (MPRAGE) T1-weighted sequence (TR/TE = 1900/3.97 ms, FA = 8°, FOV = 192 mm × 192 mm, matrix = 192 × 192, slices = 192, and slice thickness = 1.0 mm). The diffusion data for each subject were obtained using a diffusion-weighted, single shot, spin-echo, EPI sequence (TR/TE = 10200/91 ms, matrix = 96 × 96, FOV = 192 × 192 mm, voxel size = 2.0 × 2.0 × 2.0 mm^3^, 70 axial slices, 2.0 mm slice thickness, *b* value = 1500, 64 directions, and phase encoding AP). Resting-state fMRI images were collected with an echo planar imaging (EPI) sequence (TR/TE = 2450/30 ms, flip angle: 90°, FOV = 192 mm × 192 mm, matrix = 64 × 64, slice thickness = 3 mm, slices = 44, GRAPPA = 2, and 220 volumes).

### 2.5. Surface-Based Morphology Analysis (SBM)

The structural MRI was analyzed using FreeSurfer (version 5.2.0, http://surfer.nmr.mgh.harvard.edu). The details are described elsewhere [24], and it has been proven to have good accuracy in detecting cortical and subcortical structures [25, 26]. In short, the primary preprocessing includes Talairach transformation, removal of the nonbrain tissue, and segmentation of the grey matter/white matter (GM/WM) tissue. Cortical thickness is the averaged linking distance between the pial and white surfaces along normal vector. Surface area is the total area of triangles that were connected to the vertex. Volume is quantified by cortical thickness and surface area. Moreover, the entire cerebral cortex was parcellated, and a variety of surface-based data, including maps of cortical volume and surface area, were created. Data were resampled onto the FreeSurfer's average surface map according to cortical folding patterns. The cortical map of each participant was smoothed using a 10 cm full-width half-maximum (FWHM) Gaussian spatial smoothing kernel. The subcortical volumes were obtained from the automated procedure for volumetric measures of the brain structures implemented in FreeSurfer [27]. Totally, five subcortical structures (thalamus, caudate, putamen, hippocampus, and amygdala) were extracted and compared between LDH patients and HCs. We performed one-way analyses of covariance (ANCOVA) to detect subcortical volume differences between the two groups after adding the age, gender, and total brain size as covariates [28, 29]. The significant level was set at *p* < 0.05.

### 2.6. Tract-Based Spatial Statistics (TBSS)

Voxel-wise tract-based spatial statistics of diffusion-weight data was analyzed using TBSS, part of the FMRIB Software Library (FSL) (https://fsl.fmrib.ox.ac.uk/fsl/fslwiki/TBSS) [30]. This method identifies a core white matter “skeleton” that is anatomically equivalent across participants. Diffusion data preprocessing include corrections for head movement and eddy currents and brain extraction [31]. Fractional anisotropy (FA) images were created by fitting a tensor model using DTI fit, and then, FA data from all participants were aligned into a common standard space using the nonlinear registration tool FNIRT. Next, the mean FA image was created and thinned to create a mean FA skeleton representing the centers of all tracts common to the group. As Smith et al. suggested, a threshold of FA > 0.2 was applied to the skeleton with the aim of removing voxels of low FA including peripheral small tracts, where there may be high intersubject variability and gray matter [30]. We also controlled the variable of age, gender, and mean FA value by adding them as covariates. A nonparametric permutation testing with 5000 permutations was applied in the TBSS analysis using *Randomise* function of FSL. Threshold-free cluster enhancement (TFCE) with *p* < 0.05 as significant was used to obtain cluster-based statistics corrected for multiple comparisons, a method to enhance the cluster-like structures in the voxel-based data [32, 33]. Significant regions after TFCE correction *p* < 0.05 were thickened for visualization using the TBSS fill script in FSL. The Johns Hopkins University ICBM-DTI-81 white matter labels atlas was used to locate anatomical structures in the MNI152 space.

### 2.7. Resting-State fMRI Data Preprocessing

Resting-state fMRI image processing and data analyses were performed using FMRI Expert Analysis Tool (FEAT), version 5.98, which is part of the Functional Magnetic Resonance Imaging of the Brain (FMRIB) Software Library (FSL; http://www.fmrib.ox.ac.uk/fsl/). Preprocessing of resting-state fMRI data includes motion correction using MCFLIRT [34], distortion correction with field map (FUGUE), removal of nonbrain structures using Brain Extraction Tool [31], spatial smoothing using a Gaussian kernel with a 5 mm FWHM, and high-pass temporal filtering (cutoff: 100 s). Time-series autocorrelation was performed using FMRIB's Improved Linear Model (FILM). Each participants' functional MRI image was coregistered to structural image using a boundary-based registration tool [35]. Then, structural image was normalized to a standard template (MNI152-2 mm) using a linear registration (FLIRT) [34], followed by a nonlinear registration (FNIRT).

The fMRI data underwent a 2-step quality checking method by researchers. First, data were excluded if it were of poor quality due to movement (>1 mm). Second is the warp distortion amount for BBR-based function-to-structure realignment as measured by the minimal cost of the head motion as measured by the root mean square of frame-wise displacement. Three participants (two for LDH patients and one for HCs) were excluded because of head movement during the fMRI scan. In addition, four patients were excluded because of illness during scanning.

### 2.8. Seed-Based Functional Connectivity Analysis

The thalamus was chosen as a seed for the resting-state functional connectivity analyses, and the seed was identified based on the Harvard Oxford subcortical structural atlas (90% threshold), which are population-based probability atlas in MNI-152 standard space [36]. The mask was first transformed into individual functional space via inverted registration files using applywarp. Average BOLD time courses from the seed region for each individual were extracted using standard methods.

Voxel-wise seed-based functional connectivity analysis was completed using standard methods with a general linear model (GLM) framework [37]. Nuisance regions of interest for CSF and WM were generated for each subject. The mean time series of each individual's seed was set as a connectivity EV with realignment parameters, and the signals of CSF and WM were set as nuisance regressors. The parameter estimates and variances served as input to group analysis in FSL's feat using a mixed-effect FLAME approach (FLAME, FMRIB's Local Analysis of Mixed effects). The group-level statistical images were corrected using parametric family-wise error (FWE) method at cluster level, determined by *Z* > 2.3, and a corrected cluster significance threshold of *p* < 0.05. A two-group unpaired *t*-test was used for comparing differences in the thalamus-based functional connectivity between LDH patients and HCs. The group-level statistical images were corrected using parametric family-wise error (FWE) method at cluster level, determined by *Z* > 2.3, and a corrected cluster significance threshold of *p* < 0.05.

Additionally, thalamus has complex substructures, and we also performed seed-based functional connectivity analyses for different subregions of thalamus. We adopt the probability atlas of 7 subthalamic regions provided by FMRIB, which were segmented according to their white matter connectivity to cortical regions. Two different subregions of thalamus were selected as seeds for the resting-state functional connectivity analyses, which mainly connect the prefrontal and the somatosensory regions, respectively.

### 2.9. Mediation Analysis

The functional connectivity between thalamus and DLPFC was identified to be significantly related to SF-MPQ as well as BDI ratings. Therefore, to further investigate the relationship between chronic pain intensity, depression, and thalamus-DLPFC functional connectivity, a bootstrapped mediation analysis was finally employed. The mediation analyses were performed using the SPSS (IBM, version 23.0.0) version of the PROCESS macro (http://www.processmarcro.org, version 2.16.3). We tested two models: (1) independent variable was SF-MPQ, BDI was the dependent variable, and functional connectivity of thalamus-DLPFC was the mediator; (2) independent variable was BDI, SF-MPQ was the dependent variable, and functional connectivity of thalamus-DLPFC was the mediator. These analyses determined the indirect effects of thalamus-DLPFC functional connectivity on chronic pain and depression, yielding the 95% confidence intervals (CIs) of the indirect effects. A significant mediation occurs when the 95% confidence intervals did not include zero [38].

## 3. Results

### 3.1. Psychophysics

**Figure 1:**
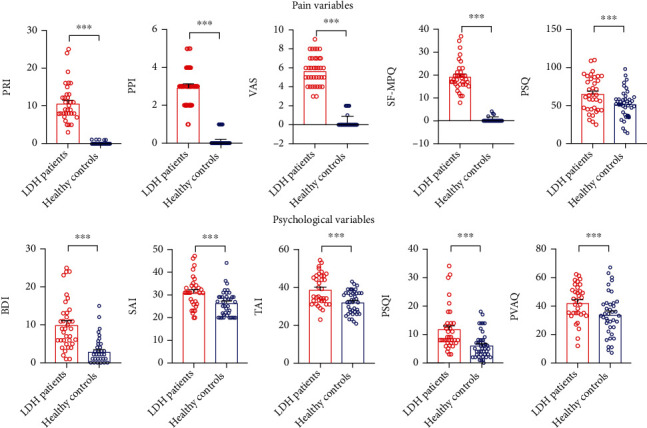
Comparison of pain intensities (i.e., PRI, PPI, VAS, and SF-MPQ), pain sensitivity (PSQ), and psychological variables (i.e., BDI, SAI, TAI, PSQI, and PVAQ) between LDH patients and HCs. (a) Pain intensity (i.e., PRI, PPI, VAS, and SF-MPQ) and pain sensitivity ratings (PSQ) were significantly larger in LDH patients than in HCs. PRI: a pain rating index; PPI: a present pain intensity; VAS: 10 cm visual analogue scale; SF-MPQ: short form of the McGill Pain Questionnaire; PSQ: pain sensitivity questionnaire. (b) Psychophysical variables (i.e., BDI, SAI, TAI, PSQI, and PVAQ) were significantly larger in LDH patients than in HCs. BDI: Beck-Depression Inventory; SAI: state-anxiety index; TAI: trait-anxiety index; PSQI: Pittsburgh sleep quality index; PVAQ: pain vigilance and awareness questionnaire; LDH: lumbar disc herniation.

The comparison of demographic, pain, and psychological characteristics between LDH patients and HCs is presented in Table 1. The age, gender, and education levels were well matched between the two groups. As expected, the intensity and sensitivity of pain, as quantified by PRI (*t* = 13.228, *p* < 0.001), PPI (*t* = 16.651, *p* < 0.001), VAS (*t* = 19.858, *p* < 0.001), total SF-MPQ scores (*t* = 17.828, *p* < 0.001), and PSQ score (*t* = 2.485, *p* = 0.015) were significantly larger in LDH patients than in HCs (Table 1 and Figure 1(a)). Furthermore, patients reported higher levels of depression (*t* = 5.866, *p* < 0.001) as well as state and trait anxiety (*t* = 3.395, *p* = 0.001; *t* = 3.985, *p* < 0.001; Table 1 and Figure 1(b)). In addition, sleep quality (PSQI, *t* = 3.893, *p* < 0.001) and pain vigilance (PVAQ, *t* = 2.461, *p* = 0.016) were significantly higher in LDH patients than in HCs (Table 1 and Figure 1(b)). Characteristics of patients with LDH are shown in Supplementary Table S1.

### 3.2. Relationship between Pain Intensities and Psychophysical Variables

**Figure 2:**
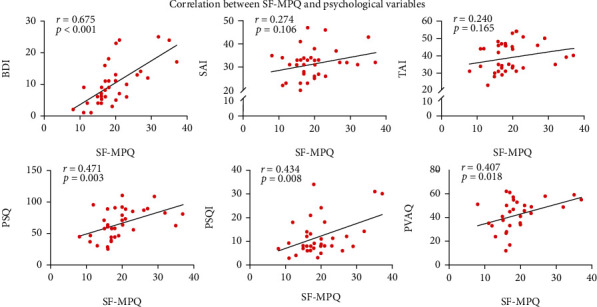
Correlation between pain intensities and psychological variables in LDH patients. (a) SF-MPQ ratings were significantly correlated with BDI, but not with SAI and TAI in LDH patients. SF-MPQ: short form of the McGill Pain Questionnaire; BDI: Beck-Depression Inventory; SAI: state-anxiety index; TAI: trait-anxiety index. (b) SF-MPQ ratings were significantly correlated with PSQ, PSQI, and PVAQ in LDH patients. PSQ: pain sensitivity questionnaire; PSQI: Pittsburgh sleep quality index; PVAQ: pain vigilance and awareness questionnaire.

For LDH patients, BDI ratings were significantly positively related with SF-MPQ ratings (*r* = 0.675, *p* < 0.001, Figure 2(a)), while both SAI and TAI ratings were not significantly related with SF-MPQ ratings (SAI vs. SF-MPQ: *r* = 0.274, *p* = 0.106 and TAI vs. SF-MPQ: *r* = 0.240, *p* = 0.165, Figure 2(a)). In addition, PSQ, PSQI, and PVAQ ratings were significantly positively correlated with SF-MPQ ratings in LDH patients (PSQ vs. SF-MPQ: *r* = 0.471, *p* = 0.003; PSQI vs. SF-MPQ: *r* = 0.434, *p* = 0.008; and PVAQ vs. SF-MPQ: *r* = 0.407, *p* = 0.018, Figure 2(b)).

### 3.3. Multiple Linear Regression Model of Clinical Pain Predictors in LDH Patients

For LDH patients, multiple linear regression analysis revealed that the dependent variable SF-MPQ was significantly influenced by the explanatory variable of BDI (accounting for 47.90% of the variability; standardized *β* = 0.704, *t* = 5.520, and *p* < 0.001), whereas not significantly affected by the explanatory variable of SAI (standardized *β* = 0.101, *t* = 0.766, and *p* = 0.450), TAI (standardized *β* = −0.215, *t* = 1.473, and *p* = 0.151), PSQI (standardized *β* = 0.234, *t* = 1.613, and *p* = 0.117), and PVAQ (standardized *β* = 0.152, *t* = 1.099, and *p* = 0.280).

### 3.4. Gray Matter Volume and Tract-Based Spatial Statistics

No significant differences in cortical and subcortical volumes were detected between LDH patients and HCs after adding the age, gender, and total brain size as the controlled variables. Subcortical volume comparisons between the two groups are presented in Supplementary Table S2.

**Figure 3:**
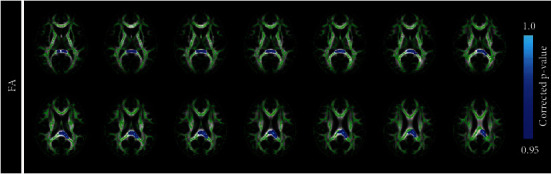
Tract-based spatial statistics maps are shown the FA differences between the two groups, and blue represents regions with significantly decreased FA in LDH patients, compared with HCs.

Compared with HCs, FA values in the region of posterior corona radiate (PCR) were significantly smaller in LDH patients (Figure 3). As chronic pain was obviously predicted by depression in LDH patients, we further conducted correlation analyses among pain intensity, depression, and FA abnormalities in the patient group. No significant correlations were observed between FA values and SF-MPQ (*r* = −0.274, *p* = 0.106) as well as BDI ratings (*r* = −0.236, *p* = 0.165) in LDH patients.

### 3.5. Seed-Based Functional Connectivity

**Figure 4:**
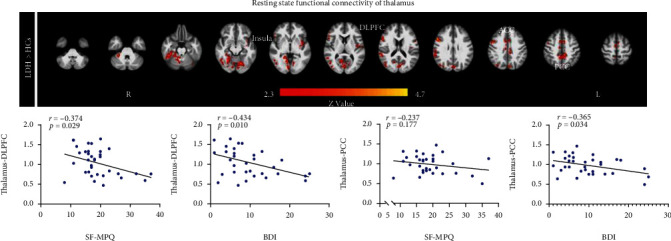
Resting-state functional connectivity of the thalamus and correlations between thalamus-based functional connectivity and SF-MPQ as well as BDI ratings. (a) Thalamus showed increased resting-state functional connectivity with the DLPFC, ACC, PCC, and insula in LDH patients than in HCs. (b) For LDH patients, resting-state functional connectivity between thalamus and DLPFC was negatively correlated with SF-MPQ and BDI ratings, and resting-state functional connectivity between thalamus and PCC was negatively correlated with BDI, but not with SF-MPQ ratings. DLPFC: dorsolateral prefrontal cortex; ACC: anterior cingulate cortex; PCC: posterior cingulate cortex; SF-MPQ: short form of the McGill Pain Questionnaire; BDI: Beck-Depression Inventory.

When the thalamus was used as the seed, resting-state functional connectivity demonstrated that thalamus exhibited stronger functional connectivity with the DLPFC, anterior cingulate cortex (ACC), insula, and posterior cingulate cortex (PCC) in LDH patients than in HCs (*Z* > 2.3, *p* < 0.05 cluster-wise corrected, Figure 4(a)). Further, we extracted abnormal thalamus-based functional connectivity and conducted correlation analyses to assess their relationship with SF-MPQ and BDI ratings in LDH patients. Results indicated that thalamus and DLPFC coupling was negatively correlated with both SF-MPQ and BDI ratings (SF-MPQ: *r* = −0.374, *p* = 0.029 and BDI: *r* = −0.434, *p* = 0.010, Figure 4(b)); thalamus and PCC coupling was negatively correlated only with BDI ratings (*r* = −0.365, *p* = 0.034), but not with SF-MPQ ratings (*r* = −0.217, *p* = 0.177, Figure 4(b)). Additionally, functional connectivity between thalamus and insula and between thalamus and ACC was negatively correlated with BDI ratings, but with a marginal significance (thalamus-insula vs. BDI: *r* = −0.329, *p* = 0.058 and thalamus-ACC vs. BDI: *r* = −0.293, *p* = 0.093), whereas functional connectivity between thalamus and insula and between thalamus and ACC was not correlated with SF-MPQ ratings (thalamus-insula vs. SF-MPQ: *r* = −0.169, *p* = 0.338 and thalamus-ACC vs. SF-MPQ: *r* = −0.248, *p* = 0.171). In addition, the results of the resting-state functional connectivity of subregions for the thalamus are presented in Supplementary Fig. S1.

### 3.6. Mediation Analysis

**Figure 5:**
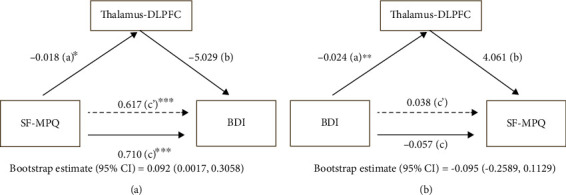
Mediation analysis. (a) The thalamus-DLPFC temporal coupling mediated the relationship from SF-MPQ to BDI. Path c is the total effect of SF-MPQ on BDI; path c' is the direct effect of SF-MPQ on BDI after controlling for the thalamus-DLPFC functional connectivity; the product of a and b (ab) is the indirect effect of SF-MPQ through the thalamus-DLPFC functional connectivity on BDI. (b) In contrast, the thalamus-DLPFC temporal coupling did not mediate the relationship from BDI to SF-MPQ. Path c is the total effect of BDI on SF-MPQ; path c' is the direct effect of BDI on SF-MPQ after controlling for the thalamus-DLPFC functional connectivity; the product of a and b (ab) is the indirect effect of BDI through the thalamus-DLPFC functional connectivity on SF-MPQ. SF-MPQ: short form of the McGill Pain Questionnaire; BDI: Beck-Depression Inventory. DLPFC: dorsolateral prefrontal cortex. ^∗^*p* < 0.05, ^∗∗^*p* < 0.01, and ^∗∗∗^*p* < 0.001.

Given that thalamus-DLPFC functional connectivity is correlated with both pain and depression ratings, we next tested the indirect effects of thalamus-DLPFC functional connectivity on chronic pain and depression. The thalamus-DLPFC temporal coupling mediated the relationship from SF-MPQ to BDI (direct effect = 0.617, *p* < 0.001; indirect effect = 0.092, 95% confidence interval: [0.002, 0.306], Figure 5(a)). In contrast, the thalamus-DLPFC temporal coupling did not mediate the relationship from BDI to SF-MPQ (direct effect = 0.038, *p* > 0.05; indirect effect = −0.095, 95% confidence interval: [-0.259, 0.113], Figure 5(b)). These results suggest that the thalamus-DLPFC coupling plays an important role in the modulation of chronic pain, possibly affecting individuals' perception of pain through regulating their depression levels.

## 4. Discussion

LDH is a chronic pain syndrome that is mainly caused by the degeneration of the lumbar disc annulus or the external pressure force burdened on the disc. The mechanism of the underlying thalamic pathway regulation of chronic pain and psychological effects in cLBP caused by lumbar disc herniation (LDH) is poorly addressed. In the present study, we investigated the relationship between brain structural/functional plasticity and the degree of chronic pain as well as pain-related psychological factors in LDH patients, and our findings can be summarized as follows: (1) LDH patients exhibited severe psychophysical disturbs (i.e., depression and anxiety), and depression was found to be an outstanding significant factor to predict chronic pain; (2) we did not observe significant structural plasticity changes for LDH patients, in terms of cortical thickness and subcortical volumes. FA values in LDH patients were identified to be significantly decreased only in the region of posterior corona radiate (PCR) but not correlated with chronic pain or any psychological factors; (3) the main finding of this study is that the functional connectivity between the thalamus and DLPFC was significantly correlated with the subjective ratings of SF-MPQ and BDI in LDH patients, and critically, the thalamus-DLPFC coupling mediated the relationship from chronic pain to depression. Our results suggested that thalamic pathways underlying prefrontal cortex might play a key role in regulation chronic pain and depression of the pathophysiology of LDH and may lead to optimized treatments in clinical practice.

### 4.1. Association between Chronic Pain and Psychophysical Characteristics in LDH

In addition to the primary etiological cause, LDH, as a typical chronic low back pain, has profound and prolonged psychophysiological consequences, such as increased depression, as demonstrated by the analysis of the multiple linear regression. Of note, different types of chronic pain conditions have been well described in a large of literature to be comorbid with psychological disorders [39]. Indeed, chronic pain and depression are commonly coexisting, and inevitably common mechanisms have been proposed. In the current study, we found that depression is the outstanding significant factor to predict chronic pain, while other psychophysical characteristics (i.e., SAI, TAI, PSQI, and PVAQ) are not significant. Depression, which was considered to be comorbidity of chronic pain, could aggravate the severity of pain during its chronicity process [14]. In clinical applications, the difficulty of relieving pain without eliminating its comorbidities has become a broad consensus. Our results emphasized the complexity of LDH, which would pose new challenges for the comprehensive assessment and accurate diagnosis of this chronic pain.

### 4.2. Structural Plasticity and Its Relationship with Psychophysical Characteristics in LDH

Structural morphology analyses demonstrated that cortical and subcortical gray matter volumes were comparable between the two groups. In addition, we assessed microstructure properties by using the voxel-wise tract-based spatial statistics method. FA is the most frequently used parameter in DTI studies, and it reflects structural integrity and geometry of axonal fibers [40]. And a different pattern in LDH patients compared with HCs was present that decreased FA values was observed in the region of PCC in LDH. Lower FA was proposed to be correlated with local cerebral edema, cerebrospinal fluid, compromised myelin structure, changes in axonal morphologic structure, and altered interaxonal spacing of fiber bundles [41]. Reduced FA values in the PCC suggest some degree of demyelination, inflammation, edema, or changes in axon count, density, diameter, or degree of crossing [42]. Since PCC is associated with pain perception, our data suggest that several aspects of pain processing and regulation may be affected in LDH. Unfortunately, we did not find significant correlations between FA values and SF-MPQ as well as BDI ratings in LDH patients. These results suggest that the chronic pain and negative psychological aspect development of LDH might not have major influence on the structural plasticity of the brain.

### 4.3. Alterations of Thalamus Connectivity and Its Relationship with Psychophysical Characteristics in LDH

Compared to HCs, LDH patients exhibited a significantly greater resting-state functional connectivity between thalamus with several brain regions, including DLPFC, ACC, insula, and PCC. Thalamus is a main gateway of nociceptive inputs to the cerebral cortex, and deficits of this region may be a reason for generalized sensory abnormalities commonly related to chronic pain [43]. Henderson et al. identified altered functional connectivity between the thalamus and cortical regions including S1, S2, and anterior insula in chronic pain patients, suggesting that chronic pain is associated with altered thalamic activity [9, 11]. The frontal lobe is an important structure involved in pain, and this structure involves the modulation of pathological algesthesia in the formation input and central sensitization [44]. Moreover, we demonstrated that the strength of temporal coupling between the thalamus and DLPFC mediated the relationship from chronic pain to depression. In consistent with previous publications, in which thalamus showed stronger resting-state function connectivity with frontal cortex when the intensity of chronic pain increased, our observations also suggested that the deficits of the ascending pain modulation system were highly associated with the intensity of chronic pain and its emotional comorbidity of depression [45].

### 4.4. Association between Chronic Pain and Depression Was Mediated by the Thalamus-DLPFC Functional Connectivity in LDH

Previous studies of chronic pain have reported consistent findings with respect to the assocaitions between clinical pain and negative emotional symptoms as well as thalamus-related functional connectivity [5, 11]. Clinical pain and negative emotional symptoms are typically defined in terms of clinical assessments. Especially, mediation analyses revealed the modulation of SF-MPQ on BDI was mediated by the thalamus-DLPFC coupling, while the modulation of BDI on SF-MPQ was not. The thalamus is the main gateway to the cerebral cortex, relaying information to specific cortical regions. Approximately 25% of the spinothalamic tract fibers terminate in the medial thalamus and then project mainly to the cingulate cortex and prefrontal cortex [46]. Several lines of evidence suggest a role for the DLPFC in the suppression of pain and maintenance of pain inhibition [47, 48]. Brascher et al. revealed that uncontrollable pain lead to increased activation of pain-related regions including the thalamus, but that DLPFC had increased negative connectivity strength during controllable pain to the thalamus, suggesting the DLPFC suppressed thalamus activity and reduced pain sensitization associated with uncontrollable pain [49]. In line with previous reports, our observations suggested that enhanced thalamus-DLPFC coupling might generate a pain suppression, thereby reducing the emotional comorbidity of depression. In addition, the DLPFC is also involved in cognitive components of the pain experience while the mediodorsal thalamus plays a role in the affective dimension of pain [48, 50, 51], and the link between chronic pain and the DLPFC-thalamus coupling could reflect persistent attempts to regulate pain. It is well documented that pain is a major risk factor for depression, and depression can exacerbate chronic pain progression [39]. Our data suggests that clinical pain affected depression indirectly through thalamus-DLPFC functional connectivity, which implies that the degree to which chronic pain states alter normal function of these circuits depends on the severity of pain in a given patient. Otherwise, the modulation of depression on pain could be attributed to different neural systems.

### 4.5. Limitations

First, the sample size of patients is limited and involves patients with varying severity and duration of disease, which limited the investigation of the detailed role of each mechanism in the development of LDH. Second, only longitudinal studies in larger samples allow us to track the development of the key variables involved over time to shed light on their relationship and develop a causal model between pathopsychophysiological factors and chronic pain severity in LDH. Third, the sample size involving LDH patients is relatively small, which limits our ability to explore in different thalamic nuclei, so that it cannot provide a precise description of the thalamocortical abnormality in LDH patients. Future research using large sample data from ultrahigh-field imaging to dissect the functional neuroanatomy of the thalamus into its components will help determine possible differences in thalamic function related to chronic pain between LDH patients and HCs. Finally, it is not clear whether LDH patients were characterized with possible transmission mechanism from the periphery to the spinal cord, which needs to be elucidated by future work with combined spinal-brain fMRI.

### 4.6. Further Prospects

Despite the limitations, this study provides further evidence that the modulation of chronic pain on depression was mediated by enhanced functional connectivity between thalamus and DLPFC. Future studies still need large sample sizes or longitudinal studies to replicate and generalize these results across large chronic pain patients. More detailed insights into the structural and functional brain plasticity underlying LDH will shed light on the development of new more targeted treatment options in clinical practice.

## Figures and Tables

**Table 1:** Demographic and pain characteristics between LDH patients and HCs (mean ± SD).

	LDH patients (36)	HCs (38)	*χ* ^2^/*t* value	*p* value
	Mean ± SD	Mean ± SD		
Female/male	11/25	10/28	0.163	0.686
Age, year	45.11 ± 10.57	43.68 ± 11.86	0.545	0.588
Education, year	11.44 ± 4.18	13.00 ± 3.13	1.818	0.073
PRI	10.67 ± 4.93	0.07 ± 0.27	13.228	<0.001
PPI	2.97 ± 0.97	0.15 ± 0.37	16.651	<0.001
VAS	5.72 ± 1.56	0.26 ± 0.64	19.858	<0.001
SF-MPQ	19.36 ± 6.41	0.50 ± 1.20	17.828	<0.001
PSQ	66.69 ± 23.25	53.79 ± 21.30	2.485	0.015
BDI	10.03 ± 6.58	2.92 ± 3.44	5.866	<0.001
SAI	31.28 ± 6.57	26.63 ± 5.49	3.395	0.001
TAI	38.83 ± 8.12	32.16 ± 6.11	3.985	<0.001
PSQI	11.86 ± 7.71	6.16 ± 4.59	3.893	<0.001
PVAQ	42.24 ± 12.63	34.03 ± 15.13	2.461	0.016

PRI: a pain rating index; PPI: a present pain intensity; VAS: 10 cm visual analogue scale; SF-MPQ: short form of the McGill Pain Questionnaire; PSQ: pain sensitivity questionnaire; BDI: Beck-Depression Inventory; SAI: state-anxiety index; TAI: trait-anxiety index; PSQI: Pittsburgh sleep quality index; PVAQ: pain vigilance and awareness questionnaire; LDH: lumbar disc herniation; HCs: healthy controls.

## Data Availability

The data and codes that support the findings of this study are available from the corresponding author upon reasonable request. The data are not publicly available due to privacy or ethical restrictions.

## Supplementary Materials

Supplementary Materials Table S1: characteristics of LDH patients. Table S2: comparison of subcortical volumes between LDH patients and HCs (mean ± SD). Fig.S1: resting-state functional connectivity of the subregions for thalamus.
