# Supplementary material for: Enhanced Temporal Coupling between Thalamus and Dorsolateral Prefrontal Cortex Mediates Chronic Low Back Pain and Depression
Source: Neural Plast. 2021 Oct 8;2021:7498714. doi: 10.1155/2021/7498714 (PMC8519723; doi:10.1155/2021/7498714)
Supplement: Supplementary Materials — Table S1: characteristics of LDH patients. Table S2: comparison of subcortical volumes between LDH patients and HCs (mean ± SD). Fig.S1: resting-state functional connectivity of the subregions for thalamus. [file 7498714.f1.docx]

**Table** **S1. Characteristics of LDH patients.**

| **Participant** | **Age** | **Sex** | **Duration of pain (month)** | **Location** | **Medication** |
| --- | --- | --- | --- | --- | --- |
| 1 | 60 | Male | 60 | Bilateral L4-L5 | Reserpine |
| 2 | 40 | Male | 36 | Left L5-S1 | - |
| 3 | 32 | Male | 12 | Left | - |
| 4 | 65 | Male | 36 | Left | Reserpine |
| 5 | 52 | Male | 120 | Left | Reserpine |
| 6 | 32 | Male | 4 | Right L5-S1 | - |
| 7 | 55 | Male | 36 | Left L4-L5 | Lbuprofen |
| 8 | 43 | Female | 3 | Left L3-L3/L5-S1 | - |
| 9 | 45 | Female | 120 | Bilateral | - |
| 10 | 52 | Male | 3 | Left | - |
| 11 | 38 | Female | 96 | Left L5-S1 | Methylcobalamin tablets |
| 12 | 39 | Female | 24 | Right | - |
| 13 | 40 | Male | 36 | Right L5 | - |
| 14 | 66 | Male | 120 | Right | - |
| 15 | 31 | Male | 3 | Left L4-L5 | - |
| 16 | 48 | Male | 3 | Left | - |
| 17 | 44 | Male | 48 | Right | - |
| 18 | 66 | Male | 4 | Bilateral L3-L4/L4-L5/L5-S1 | Metformin |
| 19 | 43 | Female | 60 | Right L4-L5 | - |
| 20 | 39 | Female | 12 | Left L4-L5/L5-S1 | Physiotherapy |
| 21 | 56 | Male | 108 | Left L4-L5/L5-S1 | - |
| 22 | 35 | Male | 12 | Left L4-L5 | - |
| 23 | 52 | Female | 48 | Left L3-L4/L4-L5/L5-S1 | Physiotherapy |
| 24 | 43 | Female | 84 | Right L4-L5/L5-S1 | Methylcobalamin tablets |
| 25 | 55 | Male | 48 | Right | Physiotherapy |
| 26 | 53 | Female | 48 | Left L5-S1 | - |
| 27 | 32 | Female | 180 | Right L4-L5/L5-S1 | Physiotherapy |
| 28 | 44 | Male | 120 | Right L4-L5 | - |
| 29 | 48 | Male | 6 | Left L3-L4/L4-L5/L5-S1 | Physiotherapy |
| 30 | 58 | Female | 48 | Right L4-L5 | Physiotherapy |
| 31 | 30 | Male | 120 | Right L4-L5/L5-S1 | - |
| 32 | 32 | Male | 12 | Left L5-S1 | Physiotherapy |
| 33 | 38 | Male | 60 | Right L5-S1 | Physiotherapy |
| 34 | 30 | Male | 36 | Left L5-S1 | Physiotherapy |
| 35 | 48 | Male | 120 | Right L4-L5/L5-S1 | Physiotherapy |
| 36 | 45 | Male | 24 | Right L4-L5/L5-S1 | Lbuprofen/Metformin |

|  | **Subcortical volume (mm^3^)** | |  |  |  |
| --- | --- | --- | --- | --- | --- |
| **Region** | **LDH patients** | **HCs** | **F** | **p** | **η_p_^2^** |
| Thalamus | 14282.0±1552.6 | 14134.9±1222.4 | 0.317 | 0.729 | 0.009 |
| Caudate | 6988.1±995.4 | 6788.1±762.3 | 0.096 | 0.909 | 0.003 |
| Putamen | 9946.7.5±1158.4 | 10150.6±1206.8 | 0.328 | 0.721 | 0.010 |
| Hippocampus | 8214.7±872.5 | 8032.4±579.5 | 0.700 | 0.500 | 0.020 |
| Amygdala | 3235.4±424.1 | 3227.9±307.1 | 0.232 | 0.793 | 0.007 |

**Table S2 Comparison of subcortical volumes between LDH patients and HCs (mean ± SD).**

LDH, lumbar disc herniation; HCs, healthy controls.

******

**Fig.S1. Resting-state functional connectivity of the sub-regions for thalamus.** (A) 7 sub-thalamic regions provided by FMRIB, which were segmented according to their white matter connectivity to cortical regions. Two different sub-regions of thalamus were chosen as seeds for the functional connectivity analyses, which mainly connect the prefrontal and the somatosensory regions respectively. (B) When region-1 was used as the seed, region-1 exhibited stronger resting-state functional connectivity with the DLPFC, insula, and ACC in LDH patients than in HCs; and resting-state functional connectivity between thalamus and DLPFC was negatively correlated with BDI ratings (r = - 0.342, p = 0.020), but not with SF-MPQ ratings (r = -0.187, p = 0.218). When region-2 was used as the seed, region-2 exhibited stronger resting-state functional connectivity with the insula, S2, ACC, and PCC in LDH patients than in HCs. DLPFC, dorsolateral prefrontal cortex; ACC, anterior cingulate cortex; PCC, posterior cingulate cortex; S2, secondary somatosensory cortex.
